# The impact of earthquakes on women: assessing women's mental health in aftermath of the Kahramanmaraş-centred earthquake in Türkiye

**DOI:** 10.1093/pubmed/fdae059

**Published:** 2024-05-03

**Authors:** Veysel Kaplan, Muhammad Alkasaby, Mehmet Emin Düken, Özlem Kaçkin, Abanoub Riad

**Affiliations:** Faculty of Health Sciences, Nursing Department, Harran University, Şanlıurfa, 63300, Turkey; Centre for Global Mental Health, London School of Hygiene & Tropical Medicine, London, WC1E 7HT, UK; Faculty of Health Sciences, Nursing Department, Harran University, Şanlıurfa, 63300, Turkey; Faculty of Health Sciences, Nursing Department, Harran University, Şanlıurfa, 63300, Turkey; Department of Public Health, Faculty of Medicine, Masaryk University, Brno, 62500, Czech Republic

**Keywords:** earthquakes, mental health, women’s health

## Abstract

**Background:**

Earthquakes disproportionately affect women and exacerbate gender and social inequalities. This study aims to investigate the psychological impact of the earthquake in Türkiye on women and the associated factors.

**Methods:**

This is a survey-based study. We collected data from 498 women residing in cities most affected by the earthquake.

**Results:**

Participants’ mean age was 27.72 ± 5.4. Over 78% of the participants lost at least one family member, and 43.7% lost at least one child due to the earthquake. The mean average of Brief Symptom Inventory (BSI) scores was 100.8 (SD = 8.37), and the Global Severity Index was 1.9 (SD = 0.16). Regression analysis showed that higher education levels predicted poor outcomes across most BSI dimensions. Losing a family member and shelter and injury status were also predictors for several mental health outcomes of the BSI subscales.

**Conclusions:**

Earthquakes significantly impact women’s well-being and may have a broader impact on the whole family. There is an urgent need to provide psycho-social interventions in the response and recovery phases of the crisis to meet the affected women’s needs. This includes providing basic needs with attention to women-specific needs, restoring social networks, addressing gender-based violence and providing gender-sensitive specialized interventions for those who need further support.

## Introduction

Earthquakes, especially major ones greater than 7.0 on the Richter scale, can cause great destruction, threaten many lives and create negative effects on the physical, mental and social development of human beings.^1^^,^^2^ Disasters triggered by natural hazards disproportionately affect different population groups. Research has shown that earthquakes can have a more significant impact on women’s well-being and mental health and that they are more likely than men to experience symptoms of depression, anxiety and post-traumatic stress.^2–4^ Research on the impact of disasters should consider that they exacerbate gender and social inequalities.^5^

On 6 February 2023, Türkiye was hit by devastating earthquakes that killed >50 000 people and displaced over 2.7 million people, 50% of whom are females.^6^ This crisis has disproportionately affected women in several ways and perpetuated pre-existing inequalities. Women face significant challenges accessing their basic needs, such as food, shelter, safety and healthcare. Even when available, services such as shelter and sanitation lack safety and privacy.^7^^,^^8^ Furthermore, an increasing number of gender-based violence (GBV), including sexual violence and harassment events, have been reported since the start of the crisis.^7^ All these factors, in addition to losing homes and loved ones and the massive destruction caused by the earthquake, make the impact of the crisis on women’s mental health inevitable.

This research aims to investigate the psychological impact of the Kahramanmaraş-centred earthquake in Türkiye on women and the associated factors. The findings of this research can inform disaster response and recovery efforts to ensure the needs of women who survived this disaster are met.

## Methods

### Study aim and design

This is a descriptive, cross-sectional study that aims to investigate the psychological impact of the earthquake on women over the age of 18 and its associated factors.

### Time, place and sample of the study

The present study was conducted 2 months after the earthquake (between 01 April 2023 and 15 May 2023) in the most affected cities: Kahramanmaraş, Hatay and Adıyaman.

The target population were women over 18 years old affected by the earthquake in these three cities. Given the logistical difficulties in obtaining a random sample of the target population, we used a convenience sampling method by collecting data from women who met the research inclusion criteria and who volunteered to participate in the study and existed in places accessible to the research team, such as temporary settlement centres, gathering areas, and aid distribution points.

### Data collection and inclusion criteria of the study

Data collection was carried out through face-to-face interviews. First, an informed consent form was shared with the participants, providing information about the study and its purpose and asking for permission to participate. Each interview took ~30 min. Women who met the following criteria were included in this study;

- willing to participate,
- over the age of 18,
- live in the three identified cities,
- can read and understand the data collection form and
- have not been diagnosed with any mental disorders.

### Data collection tool

The data collection tool consisted of the following:

*Personal Information form,* which was created by the researchers in light of the existing literature.^2^^,^^9^ Collected variables include sociodemographic data, such as age and education, in addition to variables related to the disaster, such as the number of children and family members lost due to the earthquake, the state of injury, the time spent under rubble and accommodation condition.

*Brief Symptom Inventory (BSI):* The inventory was developed by Derogatis (1992) to evaluate psychopathological and psychological symptoms.^10^ Şahin and Durak (1994) conducted a validity study of its Turkish version.^11^ The inventory has 53 items with nine subscales: anxiety, depression, somatization, hostility, obsession–compulsion, interpersonal sensitivity, phobic anxiety, paranoid ideation and psychoticism. Each subscale is represented by a number of items in the BSI. Items are scored on a 5-point scale as follows: 0 (not at all), 1 (slightly), 2 (moderately), 3 (very) and 4 (extremely). The highest possible score is 212, and the lowest is 0. Higher scores indicate more severe symptoms. The BSI has three global indices. The Global Severity Index (GSI) is the most sensitive indicator of the participant’s distress level. The GSI is calculated using the sums for all items divided by the total number of items to which the participant responded. The total score of the internal consistency (Cronbach’s alpha) coefficient for the Turkish version of BSI was 0.96.

### Data analysis

Data was analysed using IBM SPSS Statistics version 29.0 (IBM Corp., Armonk, NY, USA). Descriptive statistics, including percentages, medians and ranges, were used to summarize the sociodemographic characteristics, as the data were not normally distributed based on the Kolmogorov–Smirnov test. Regarding losing children, family members or the period spent under rubble, we calculated the median and range for those who positively answered those questions (i.e. the range of the number of children or family members lost and hours spent under rubble (Table 1). The total BSI score was calculated by summing the score of all 53 items, and the GSI score was calculated by summing all items’ scores divided by the total number of items to which the participant responded. We also calculated the scores of each subscale separately and presented them as median and interquartile range (IQR).

**Table 1:** Demographic characteristics of women in reproductive age from Kahramanmaraş earthquake-affected cities, April–May 2023, (*n* = 498)

Variable	Outcome	Age group		Total (*n* = 498)	*P.*
		18–30 years old (*n* = 370)	31–42 years old (*n* = 128)		
**Education**	Literate (1-5 years)	70 (18.9%)	98 (76.6%)	168 (33.7%)	**<0.001**
	Primary education(6-8 years)	80 (21.6%)	28 (21.9%)	108 (21.7%)	
	Secondary education (9-12 years)	108 (29.2%)	2 (1.6%)	110 (22.1%)	
	Higher education (≥13 years)	112 (30.3%)	0 (0%)	112 (22.5%)	
**Lost children**	No	266 (72.7%)	12 (9.4%)	278 (56.3%)	**<0.001**
	Yes	100 (27.3%)	116 (90.6%)	216 (43.7%)	
	Median (Range)	1 (1–2)	1 (1–2)	1 (1–2)	0.126
**Lost nuclear family members**	No	96 (25.9%)	12 (9.4%)	108 (21.7%)	**<0.001**
	Yes	274 (74.1%)	116 (90.6%)	390 (78.3%)	
	Median (Range)	1 (1–3)	1 (1–3)	1 (1–3)	0.345
**Being under debris**	No	12 (3.2%)	8 (6.3%)	20 (4%)	0.135
	Yes	358 (96.8%)	120 (93.8%)	478 (96%)	
	Median (Range)	18 (4–40)	24 (10–48)	20 (4–48)	**<0.001**
**Being injured**	No	66 (17.8%)	8 (6.3%)	74 (14.9%)	**0.001**
	Yes	304 (82.2%)	120 (93.8%)	424 (85.1%)	
**Shelter status**	Tents	248 (67%)	128 (100%)	376 (75.5%)	**<0.001**
	Metallic containers	116 (31.4%)	0 (0%)	116 (23.3%)	
	Other	6 (1.6%)	0 (0%)	6 (1.2%)	

Chi-squared (*χ^2^*), Fisher’s exact and Mann–Whitney (*U*) tests were used with a significance level (*P.*) <0.05. Values in Bold are statistically significant values.

In addition to the Chi-squared test and Fisher’s exact test, the non-parametric Mann–Whitney U test was used to compare median scores between two independent groups, and the Kruskal–Wallis H test was used to compare median scores between more than two independent groups. All inferential tests were conducted with <5% significance level.

Multivariable linear regression analysis was conducted to identify predictors of mental health outcomes. The dependent variables were the BSI subscale scores, and the independent variables were age group, education level, loss of a child or family member, being under debris, injury status and shelter status. Dummy coding was used for categorical variables. Standardized beta coefficients and 95% confidence intervals were reported. R-squared values were calculated to determine the variance explained by the models.

### Ethical considerations

The ethics approval was obtained from the Ethics Committee of Harran University (Protocol Number: E-76244175-050.01.04-226646). During the study process, the ethical rules stated in the Helsinki Declaration were observed, and informed consent was obtained from the participants. Before data collection, the purpose of the present study, its duration and the participant’s rights were explained. The participants were told they could withdraw from the study at any time, and that their information would be kept confidential.

## Results

Five hundred twenty-one women were reached out, and 498 of them agreed to participate in the study. The mean age of the women was 27.72 ± 5.4. Around 78.3% of the participants lost at least one family member due to the earthquake, and 43.7% lost at least one child. The older age group (over 30 years old) had significantly higher rates of losing one child or more (90.6 versus 27.3%) and nuclear family members (90.6 versus 74.1%) compared with the younger women group. Additionally, 85.1% of women were injured during the earthquake. Injuries among women over 30 years were higher (93.8%) compared with the younger women group (82.2%). Almost all women surveyed (96%) reported being under debris, but the median time women over 30 spent under debris was higher (24 hours) compared with the younger women group (18 hours). Furthermore, 75.5% of the women interviewed live in tents. Table 1 summarizes the sociodemographic and personal characteristics of the women who participated in the study.

The mean average of BSI scores was 100.8 (SD = 8.37), and the GSI was 1.9 (SD = 0.16). Table 2 shows the association between the sociodemographics and the different subscales of the BSI. There was a statistically significant association between the age of participants and obsession-compulsion, interpersonal sensitivity, depression, phobic anxiety, paranoid ideation and psychoticism subscales of BSI. However, there was no significant difference between age groups when looking at the median scores of the BSI subscales. Seemingly, there was an association between the status of injury (injured or not) and some of the BSI subscales, with no significant difference in the median of all BSI subscales scores between both age groups. There was an association between education and most of the BSI subscales, especially obsession-compulsion, anxiety and phobic anxiety, where the median scores were significantly higher in participants with higher education.

**Table 2:** BSI subscales outcome among women affected by Kahramanmaraş-centred earthquake, April–May 2023, (*n* = 498)

Variable	Outcome	Somatization	*P.*	Obsession-compulsion	*P.*	Interpersonal sensitivity	*P.*
**Age group**	18–30 years old	13 (12–14)	0.832	12 (11–14)	**0.002**	8 (7–9)	**<0.001**
	31–42 years old	13 (12–14)		12 (10–13)		7 (7–8)	
**Education**	Literate	13 (12–14)	**<0.001**	12 (10–13)	**<0.001**	7 (6.25–8)	**<0.001**
	Primary education	13 (12–15)		12 (10–13)		7 (7–8)	
	Secondary education	13 (12–14)		13 (11–14)		8 (7–9)	
	Higher education (≥13)	12 (12–14)		14 (11–15)		9 (8–9)	
**Lost children**	No	13 (12–14)	0.425	13 (11–14)	**<0.001**	9 (8–9)	**<0.001**
	Yes	13 (12–14)		12 (10–13)		7 (7–8)	
**Lost nuclear family members**	No	13 (12–14)	0.279	14 (12–14)	**<0.001**	8 (8–9)	**0.004**
	Yes	13 (12–15)		12 (10–13)		8 (7–9)	
**Being under debris**	No	12.5 (12–14)	0.642	12 (10–14)	0.916	8 (8–9)	0.473
	Yes	13 (12–14)		12 (11–14)		8 (7–9)	
**Being injured**	No	12 (12–14.25)	0.194	13 (11–15)	**<0.001**	9 (8–9)	**<0.001**
	Yes	13 (12–14)		12 (10–13)		8 (7–9)	
**Shelter status**	Tents	13 (12–14)	**<0.001**	12 (10–13)	**<0.001**	8 (7–9)	**<0.001**
	Metallic containers	12 (11–13)		13 (11–15)		9 (8–9)	
	Other	14 (12–16)		11 (10–15)		8 (8–9)	
**Total**	**Median (IQR)**	**13 (12–14)**		**12 (11–14)**		**8 (7–9)**	
**Variable**	**Outcome**	**Depression**	***P.***	**Anxiety**	***P.***	**Hostility**	***P.***
**Age group**	18–30 years old	12 (11–13)	**<0.001**	11 (10–12)	0.617	10 (8–10)	0.062
	31–42 years old	11 (10–12)		11 (10–12)		9 (8–10)	
**Education**	Literate	11 (10–12)	**<0.001**	11 (10–12)	**<0.001**	9 (8–10)	0.139
	Primary education	11 (10–12)		11 (10–12)		9 (8–10)	
	Secondary education	12 (11–12)		11 (10–12)		10 (9–10)	
	Higher education	12 (12–15)		12 (11–12.75)		10 (7–12)	
**Lost children**	No	12 (12–13)	**<0.001**	11 (10–12)	0.363	10 (8–10)	0.130
	Yes	11 (10–12)		11 (10–12)		9 (8–10)	
**Lost nuclear family members**	No	12 (12–12)	0.611	10 (9–11)	**<0.001**	10 (9–10)	0.100
	Yes	12 (10–13)		11 (10–13)		9 (8–10)	
**Being under debris**	No	12 (11–13)	0.264	10.5 (9–11)	**0.018**	9.5 (9–10)	0.874
	Yes	12 (11–13)		11 (10–12)		9 (8–10)	
**Being injured**	No	12 (12–14.25)	**<0.001**	12 (11–13)	**<0.001**	9 (7–10.25)	0.135
	Yes	12 (10–12)		11 (10–12)		10 (8–10)	
**Shelter status**	Tents	12 (10–12)	**<0.001**	11 (10–12)	**<0.001**	9 (8–10)	0.129
	Metallic containers	12 (12–14)		12 (11–13)		10 (7–12)	
	Other	13 (10–15)		11 (11–12)		8 (7–10)	
**Total**	**Median (IQR)**	**12 (11–13)**		**11 (10–12)**		**9 (8–10)**	
**Age group**	18–30 years old	11 (9.75–11)	**<0.001**	10 (9–11)	**<0.001**	10 (9–11)	**0.050**
	31–42 years old	9 (9–10)		10 (8.25–11)		10 (9–10)	
**Education**	Literate	9 (9–10)	**<0.001**	10 (9–11)	**<0.001**	10 (9–10)	0.131
	Primary education	9.5 (8–10)		10 (9–11)		10 (9–11)	
	Secondary education	11 (10–11)		10 (9–12)		10 (9–11)	
	Higher education	11 (11–11)		11 (10–11)		10 (8.25–11)	
**Lost children**	No	11 (10–11)	**<0.001**	10 (9–11)	**<0.001**	10 (9–11)	0.600
	Yes	9 (9–10)		10 (9–11)		10 (9–10)	
**Lost nuclear family members**	No	11 (10–11)	**<0.001**	11 (9–13)	**<0.001**	9 (9–11)	0.256
	Yes	10 (9–11)		10 (9–11)		10 (9–11)	
**Being under debris**	No	9 (9–11)	0.119	11 (9–12)	0.294	10 (10–11)	0.060
	Yes	10 (9–11)		10 (9–11)		10 (9–11)	
**Being injured**	No	11 (10–11)	**<0.001**	11 (10–11)	**<0.001**	10 (9–11)	0.549
	Yes	10 (9–11)		10 (9–11)		10 (9–11)	
**Shelter status**	Tents	10 (9–11)	**<0.001**	10 (9–11)	**<0.001**	10 (9–11)	**0.037**
	Metallic containers	11 (11–11)		10.5 (10–11)		10 (8–10)	
	Other	11 (8–12)		10 (9–11)		10 (9–10)	
**Total**	**Median (IQR)**	**10 (9–11)**		**10 (9–11)**		**10 (9–11)**	

Mann–Whitney (*U*) and Kruskal–Wallis (*H*) tests were used with a significance level (*P.*) <0. Values in bold are statistically significant values.

Interestingly, the median obsession-compulsion and hostility scores were higher among those who mentioned that they did not lose a child and those who did not lose a family member compared with those who lost a child or a family member. In comparison, median anxiety and psychoticism scores were higher among those who lost a family member. It was also found that there is an association between shelter status and all BSI subscales except hostility, while being under debris was only associated with anxiety.

We conducted a multivariate regression analysis to identify the predictors of mental health outcomes among women affected by the earthquake. Regression analysis results (Table 3) showed that education level strongly predicted mental health outcomes across several BSI subscales. Women with higher education had significantly higher scores of obsession-compulsion (β = 1.291, 95% CI: 0.481–2.100), interpersonal sensitivity (β = 1.080, CI: 0.542–1.618), depression (beta = 0.880, CI 0.187 to 1.572), phobic anxiety (β = 1.055, 95% CI: 0.529–1.580) and paranoid ideation (β = 1.176, 95% CI: 0.532–1.821) compared with literate women (who have <6 years of education). Losing nuclear family members in the earthquake significantly predicted higher scores of somatization (β = 0.682, 95% CI: 0.220–1.143) and anxiety (β = 1.344, 95% CI: 0.942–1.745). Interestingly, losing a child was a predictor for lower depression (β = −0.530, 95% CI: −1.029 to −0.031) and anxiety (β = −0.514, 95% CI: −0.987 to −0.041) scores. Seemingly, Being under debris was a predictor for lower interpersonal sensitivity (β = −0.673, 95% CI: −1.290 to −0.055) scores and being injured predicted lower obsession-compulsion, depression and paranoid ideation scores in addition to higher hostility scores. The shelter status significantly predicted mental health outcomes on the BSI scale. Living in a container predicted better somatization (β = −0.757, 95% CI: −1.390 to −0.125), paranoid ideation (β = −0.609, 95% CI: −1.150 to −0.069) and psychotism (β = −0.610, 95% CI: −1.082 to −0.137) scores compared with living in tents, but it was associated with higher anxiety (β = 1.161, 95% CI: 0.612–1.711) and phobic anxiety (β = 0.475, 95% CI: 0.035–0.916) scores. After controlling for other factors, age only predicted lower phobic anxiety scores in the older age group of women (β = −0.370, 95% CI: −0.676 to 0.063).

**Table 3:** Regression models of BSI among women in reproductive age from Kahramanmaraş earthquake-affected cities, April–May 2023, (*n* = 498)

Predictor	Somatization (R^2^ = 6.28%)				Obsession-Compulsion (R^2^ = 15.7%)				Interpersonal sensitivity (R^2^ = 18.7%)			
	Beta	SE	95% CI	*P.*	Beta	SE	95% CI	*P.*	Beta	SE	95% CI	*P.*
**Age group:** 31–42 versus (18–30 years old)	−0.063	0.224	−0.503 – 0.377	0.779	−0.013	0.240	−0.485 – 0.459	0.957	−0.006	0.160	−0.320 – 0.308	0.970
**Education level:** primary versus literate	0.665	0.234	0.205 – 1.125	**0.005**	0.235	0.251	−0.258 – 0.729	0.349	0.011	0.167	−0.318 – 0.339	0.950
**Education level:** secondary versus literate	0.710	0.315	0.092 – 1.329	**0.024**	0.497	0.337	−0.166 – 1.160	0.141	0.666	0.224	0.225 – 1.107	**0.003**
**Education level:** higher versus literate	0.310	0.384	−0.444 – 1.065	0.420	1.291	0.412	0.481 – 2.100	**0.002**	1.080	0.274	0.542 – 1.618	**< 0.001**
**Lost children:** yes versus no	−0.175	0.277	−0.718 – 0.369	0.528	0.291	0.297	−0.292 – 0.874	0.328	−0.220	0.197	−0.608 – 0.167	0.264
**Lost nuclear family member:** yes versus no	0.682	0.235	0.220 – 1.143	**0.004**	−1.157	0.252	−1.652 – −0.662	**< 0.001**	0.156	0.168	−0.173 – 0.485	0.352
**Being under debris:** yes versus no	−0.090	0.441	−0.956 – 0.776	0.838	0.187	0.473	−0.742 – 1.116	0.693	−0.673	0.314	−1.290 – −0.055	**0.033**
**Being injured:** yes versus no	−0.170	0.279	−0.719 – 0.379	0.543	−0.767	0.300	−1.356 – −0.179	**0.011**	0.175	0.199	−0.216 – 0.567	0.380
**Shelter status:** metallic containers versus tents	−0.757	0.322	−1.390 – −0.125	**0.019**	0.216	0.345	−0.462 – 0.894	0.532	0.240	0.230	−0.211 – 0.691	0.297
**Shelter status:** other versus tents	0.916	0.718	−0.494 – 2.327	0.202	0.268	0.770	−1.244 – 1.780	0.728	0.569	0.512	−0.437 – 1.574	0.267
*Predictor*	*Depression (R^2^ = 22%)*				*Anxiety (R^2^ = 20.5%)*				*Hostility (R^2^ = 3.14%)*			
	*Beta*	*SE*	*95% CI*	*P.*	*Beta*	*SE*	*95% CI*	*P.*	*Beta*	*SE*	*95% CI*	*P.*
**Age group:** 31 – 42 versus (18 – 30 years old)	−0.073	0.205	−0.476 – 0.331	0.724	0.208	0.195	−0.175 – 0.590	0.287	−0.280	0.202	−0.676 – 0.116	0.166
**Education level:** primary versus literate	−0.380	0.215	−0.802 – 0.042	0.078	−0.081	0.204	−0.481 – 0.320	0.691	−0.152	0.211	−0.566 – 0.263	0.472
**Education level:** secondary versus literate	0.177	0.289	−0.391 – 0.744	0.541	−0.151	0.274	−0.690 – 0.387	0.582	0.158	0.283	−0.399 – 0.715	0.577
**Education level:** higher versus literate	0.880	0.352	0.187 – 1.572	**0.013**	−0.489	0.334	−1.145 – 0.168	0.144	0.144	0.346	−0.536 – 0.823	0.678
**Lost children:** yes versus no	−0.530	0.254	−1.029 – −0.031	**0.037**	−0.514	0.241	−0.987 – −0.041	**0.033**	0.194	0.249	−0.296 – 0.683	0.438
**Lost nuclear family member:** yes versus no	0.357	0.216	−0.067 – 0.780	0.098	1.344	0.204	0.942 – 1.745	**< 0.001**	−0.067	0.212	−0.483 – 0.349	0.751
**Being under debris:** yes versus no	−0.626	0.404	−1.421 – 0.169	0.122	0.038	0.307	−0.565 – 0.642	0.901	−0.301	0.397	−1.081 – 0.479	0.449
**Being injured:** yes versus no	−0.576	0.256	−1.080 – −0.072	**0.025**	0.024	0.195	−0.358 – 0.407	0.902	0.637	0.252	0.142 – 1.131	**0.012**
**Shelter status:** metallic containers versus tents	0.317	0.295	−0.263 – 0.897	0.284	1.161	0.280	0.612 – 1.711	**< 0.001**	0.355	0.290	−0.214 – 0.925	0.221
**Shelter status:** other versus tents	1.026	0.658	−0.268 – 2.320	0.120	0.455	0.500	−0.527 – 1.481	0.363	−1.228	0.646	−2.497 – 0.042	0.058
*Predictor*	*Phobic anxiety (R^2^ = 29.9%)*				*Paranoid ideation (R^2^ = 16.8%)*				*Psychoticism (R^2^ = 4.25%)*			
	*Beta*	*SE*	*95% CI*	*P.*	*Beta*	*SE*	*95% CI*	*P.*	*Beta*	*SE*	*95% CI*	*P.*
**Age group:** 31 – 42 versus (18 – 30 years old)	−0.370	0.156	−0.676 – 0.063	**0.018**	−0.105	0.191	−0.481 – 0.271	0.583	−0.280	0.167	−0.608 – 0.049	0.095
**Education level:** primary versus literate	−0.028	0.163	−0.349 – 0.292	0.860	−0.003	0.200	−0.396 – 0.390	0.987	0.191	0.175	−0.152 – 0.535	0.274
**Education level:** secondary versus literate	0.744	0.219	0.313 – 1.175	**< 0.001**	0.629	0.269	0.101 – 1.158	**0.020**	0.343	0.235	−0.119 – 0.805	0.145
**Education level:** higher versus literate	1.055	0.268	0.529 – 1.580	**< 0.001**	1.176	0.328	0.532 – 1.821	**< 0.001**	0.417	0.287	−0.146 – 0.981	0.146
**Lost children:** yes versus no	−0.090	0.193	−0.468 – 0.289	0.642	−0.042	0.236	−0.506 – 0.423	0.860	−0.135	0.207	−0.540 – 0.271	0.515
**Lost nuclear family member:** yes versus no	−0.206	0.164	−0.528 – 0.116	0.209	−0.884	0.201	−1.278 – −0.490	**< 0.001**	0.456	0.175	0.111 – 0.801	**0.010**
**Being under debris:** yes versus no	0.038	0.307	−0.565 – 0.642	0.901	−0.232	0.377	−0.971 – 0.508	0.539	−0.378	0.329	−1.024 – 0.269	0.252
**Being injured:** yes versus no	0.024	0.195	−0.358 – 0.407	0.902	−0.485	0.239	−0.954 – −0.016	**0.043**	−0.124	0.209	−0.534 – 0.286	0.554
**Shelter status:** metallic containers versus tents	0.475	0.224	0.035 – 0.916	**0.035**	−0.609	0.275	−1.150 – −0.069	**0.027**	−0.610	0.240	−1.082 – −0.137	**0.012**
**Shelter status:** other versus tents	0.455	0.500	−0.527 – 1.437	0.363	−0.011	0.613	−1.215 – 1.193	0.986	−0.420	0.536	−1.473 – 0.633	0.433

Values in bold are statistically significant values.

## Discussion

### Main findings of this study

This study investigated the psychological impact of the Kahramanmaraş-centred earthquake in Türkiye on women and the associated factors. The study indicates a high prevalence of psychological symptoms such as anxiety, depression and somatization among participants. The mean average of BSI scores was 100.8 (SD = 8.37), and the GSI was 1.9 (SD = 0.16). Regression analysis showed that higher education levels predicted poor outcomes across most BSI dimensions. Losing a family member and shelter and injury status were also predictors for several mental health outcomes of the BSI subscales.

### What is already known on this topic

Studies conducted after earthquakes in Türkiye, Pakistan, Iran and Nepal reported that earthquakes as traumatic events can cause several mental problems due to their widespread and devastating effects.^12–14^ The recent earthquake in Türkiye, which affected 11 provinces, killed over 50 000 and displaced millions after they lost their homes, livelihoods and loved ones. The mean GSI reported in this study (1.9 [SD = 0.16]) was significantly higher than the mean GSI reported by Altamore et al.^15^, who reported a mean GSI of 0.47 (SD = 0.47) among those affected by the 2016 central Italy earthquakes. About 60% of the participants in the previous study were men, and the age range was from 35 to 76 years. This can partially explain the significant difference between the two mean scores, as it is known that disasters triggered by natural hazards have more impact on women compared with men.^5^ Additionally, given that the study conducted in Italy collected data a year after the earthquake, psychological symptoms might have improved over time. This also can explain the high scores in our study, as it was conducted 2 months after the crisis. It is expected that a significant portion of the psychological symptoms detected in this study might disappear over time if proper support is provided.

Women affected by the earthquake showed high scores across the different BSI subscales. Somatization was one of the symptoms detected among women affected by the earthquake. It was associated with the level of education, losing a family member and shelter status, where women living in tents were predicted to have high somatization scores compared with those living in containers. This is consistent with other studies that associate exposure to earthquakes with increasing the incidence of somatic symptoms and worsening the already existing symptoms.^16^ Somatization is common in non-Western societies, where somatic symptoms can be an indicator of distress.^17^ Karaköse and Ulusoy (2022) reported that stress among Turkish housewives is mostly expressed in the form of somatic symptoms, which leads to frequent visits to health services for medically unexplained symptoms. Considering the high rate of injuries among women affected by the earthquake (over 85%) in addition to the somatic manifestations of stress, these women may frequently visit healthcare services. Therefore, it is crucial to bring the attention of healthcare workers and others involved in the earthquake response to the different signs of distress, especially the psychosomatic ones.

### What this study adds

This study identify some of the factors associated with mental health outcomes among women affected by earthquakes and provides an explanation of this impact. For example, higher educational levels predicted worse psychological outcomes among women affected by the earthquake. Women with higher education might have more established careers, income sources and better living conditions that were disrupted by the earthquake. The loss of comfort and stability, dependence on aid, and struggle to adapt to the new situation may lead to more significant distress. Additionally, these women may have higher career and life aspirations and have experienced a greater sense of loss, disruption and uncertainty when the earthquake occurred.

One of the surprising findings is that losing a child was associated with lower anxiety and depression symptoms. Women who lost a child may receive more attention, including social and psychological support, which could partially explain the low anxiety and depression levels. Social support may reduce the negative impact of disasters on mental health.^18^ In addition to its direct positive impact on mental health, social support was found to mitigate the impact of other disaster-related stressors, especially among women.^19^ Losing a child is a profound traumatic experience that may cause emotional numbness among this group of women, reducing the expression of depression, anxiety and other psychological symptoms. This may also partially explain the lower BSI subscales among women injured and those who spent hours under debris. Denial as a coping mechanism can be effective in the short term as it allows time to process the traumatic event. It was associated with positive mental health outcomes among people with chronic conditions during COVID-19.^20^ However, those women eventually have to deal with the reality and cope with the loss and massive disruption caused by the earthquake.

### Limitations of this study

The present study has a few limitations. First, data were collected only two months after the earthquakes, which might lead to pathologizing what could be considered a normal reaction to this crisis. Additionally, taking the study sample from temporary settlements established for people who lost their homes could also contribute to high psychological symptoms detected among the study sample, which might not be representative of the whole population affected by the crisis. Furthermore, due to logistical difficulties,such as having a registry that covers the target population toconduct random sampling and reaching out to this sample of women, we used non-probability sampling method, which means that the study’s results can only apply to the study participants. Additionally, this study collected only quantitative data, which does not provide a deeper understanding of the experience of women affected by the earthquake. However, this study’s findings could still be used as an indicator of the crisis’s massive impact on women’s mental health and what factors should be addressed to mitigate this impact. We recommend conducting qualitative studies to gain a deeper understanding of these women’s experience and their needs. Since we have excluded people diagnosed with a mental health condition, this study did not capture the worsening symptoms of the existing mental health conditions.

### Recommendations

To protect and improve community mental health, it is necessary to ensure that earthquake response and recovery activities meet the needs of women. In this context, we suggest the following in line with the results of this study, existing literature, and our experience as mental health professionals involved in disaster response:

- Ensure that disaster response and recovery plans are gender-responsive to meet the needs of affected girls and women.
- Ensure that psycho-social considerations are taken into account in all response activities. This, at its most basic, means ensuring that basic needs are delivered in a safe, dignified and socially appropriate way.^21^
- Prioritize the basic needs of vulnerable groups in the earthquake-affected areas, including women and children and give attention to the group-specific needs.
- Address women’s sexual and reproductive health, which might not be considered a priority in this situation. This can include distributing hygiene products and installing appropriate sanitary facilities in the camps.^22^
- GBV is more likely to increase, especially in the temporary camps created post-earthquake. Therefore, special teams should be formed, and continuous evaluations should be made to prevent GBV incidents and support GBV survivors.^22^
- Ensure emergency responders are trained in providing psychological first aid with a gender-sensitive approach.
- There has to be gender diversity among staff working in temporary accommodation, as the presence of female staff can help women feel safe.
- Conduct outreach activities to raise awareness about the psychological impact of the crisis and healthy coping mechanisms. Given the stigma around mental health, many may not seek help.
- Given the shared trauma of losing children and family members, women in the affected communities need safe spaces to come together to grieve and receive support. This also will help reconstruct social networks, which act as a protective factor against mental health conditions.
- Mental Health and Psycho-Social Support (MHPSS) activities should cover the different psychological needs of the affected women. While most women can feel better after providing basic needs and social support, some may need specialized mental health interventions (i.e. interventions provided by a trained specialist).^21^
- Psycho-social support interventions should be included in the recovery plans to promote resilience and mitigate the long-term impact of the earthquake on girls and women.

## Conclusion

This study examined the adverse psychological effects of the Kahramanmaraş-centred earthquake in 2023 among a sample of women living in the three cities. The study indicates a high prevalence of psychological symptoms such as anxiety, depression and somatization among participants. The mean average of BSI scores was 100.8 (SD = 8.37), and the GSI was 1.9 (SD = 0.16). Higher education levels predicted poor outcomes across most BSI dimensions. Losing a family member and shelter and injury status were also predicted several psychological outcomes. Our results indicate that earthquakes present a serious threat to women’s mental health and well-being, which might have a wider impact on the whole family, especially children. There is an urgent need to support and empower women affected by the earthquake throughout the response and recovery phases of the crisis by providing psycho-social interventions that cover the different levels of the Inter-agency Standing Committee (IASC) MHPSS intervention pyramid. This includes providing basic needs with attention to women- and girls-specific needs (such as hygiene products and safe sanitary facilities), reconstructing social networks, addressing GBV and providing gender-sensitive specialized interventions for those who need further support.

## Data Availability

The data underlying this article will be shared on reasonable request to the corresponding author.

## Notes

**Veysal Kaplan,** Associate professor

**Muhammad Alkasaby,** Research Fellow

**Mehmet Emin Düken,** Research Assistant

**Özlem Kaçkin,** Assistant Professor

**Abanoub Riad,** Assistant Professor
