# Correction to: The impact of earthquakes on women: assessing women's mental health in aftermath of the Kahramanmaraş-centred earthquake in Türkiye

**DOI:** 10.1093/pubmed/fdae075

**Published:** 2024-05-17

This is a correction to: Veysel Kaplan, Muhammad Alkasaby, Mehmet Emin Düken, Özlem Kaçkin, Abanoub Riad, The impact of earthquakes on women: assessing women's mental health in aftermath of the Kahramanmaras-centred earthquake in Türkiye, Journal of Public Health, 2024, fdae059, https://doi.org/10.1093/pubmed/fdae059

In the originally published version of this article, there was a typographical error in author Veysel Kaplan's name.

This has been corrected.

